# Co‐designing a theory‐informed, multicomponent intervention to increase vaccine uptake with Congolese migrants: A qualitative, community‐based participatory research study (LISOLO MALAMU)

**DOI:** 10.1111/hex.13884

**Published:** 2023-10-13

**Authors:** Alison F. Crawshaw, Lusau M. Kitoko, Sarah L. Nkembi, Laura M. Lutumba, Caroline Hickey, Anna Deal, Jessica Carter, Felicity Knights, Tushna Vandrevala, Alice S. Forster, Sally Hargreaves

**Affiliations:** ^1^ The Migrant Health Research Group, Institute for Infection and Immunity St George's University of London London UK; ^2^ Hackney Congolese Women Support Group, Hackney CVS London UK; ^3^ Hackney Refugee and Migrant Forum and Hackney CVS London UK; ^4^ Faculty of Public Health and Policy, London School of Hygiene and Tropical Medicine London UK; ^5^ Centre for Applied Health and Social Care Research, Faculty of Health, Science, Social Care and Education Kingston University London London UK; ^6^ Our Future Health, New Bailey Manchester UK

**Keywords:** behavioural psychology, community‐based participatory research, COVID‐19 vaccines, intervention development, migrants, refugees, health inequalities

## Abstract

**Introduction:**

Disparities in the uptake of routine and COVID‐19 vaccinations have been observed in migrant populations, and attributed to issues of mistrust, access and low vaccine confidence. Participatory research approaches and behaviour change theory hold the potential for developing tailored vaccination interventions that address these complex barriers in partnership with communities and should be explored further.

**Methods:**

This study used a theory‐informed, community‐based participatory research approach to co‐design a culturally tailored behaviour change intervention aimed at increasing COVID‐19 vaccine uptake among Congolese migrants in London, United Kingdom (2021–2022). It was designed and led by a community‐academic partnership in response to unmet needs in the Congolese community as the COVID‐19 pandemic started. Barriers and facilitators to COVID‐19 vaccination, information and communication preferences, and intervention suggestions were explored through qualitative in‐depth interviews with Congolese migrants, thematically analysed, and mapped to the theoretical domains framework (TDF) and the capability, opportunity, motivation, behaviour model to identify target behaviours and strategies to include in interventions. Interventions were co‐designed and tailored in workshops involving Congolese migrants.

**Results:**

Thirty‐two Congolese adult migrants (24 (75%) women, mean 14.3 (SD: 7.5) years in the United Kingdom, mean age 52.6 (SD: 11.0) years) took part in in‐depth interviews and 16 (same sample) took part in co‐design workshops. Fourteen barriers and 10 facilitators to COVID‐19 vaccination were identified; most barrier data related to four TDF domains (beliefs about consequences; emotion; social influences and environmental context and resources), and the behavioural diagnosis concluded interventions should target improving psychological capability, reflective and automatic motivations and social opportunities. Strategies included culturally tailored behaviour change techniques based on education, persuasion, modelling, enablement and environmental restructuring, which resulted in a co‐designed intervention comprising community‐led workshops, plays and posters. Findings and interventions were disseminated through a community celebration event.

**Conclusions:**

Our study demonstrates how behavioural theory can be applied to co‐designing tailored interventions with underserved migrant communities through a participatory research paradigm to address a range of health issues and inequalities. Future research should build on this empowering approach, with the goal of developing more sensitive vaccination services and interventions which respond to migrant communities' unique cultural needs and realities.

**Patient or Public Contribution:**

Patient and public involvement (PPI) were embedded in the participatory study design and approach, with community members co‐producing all stages of the study and co‐authoring this paper. An independent PPI board (St George's Migrant Health Research Group Patient and Public Involvement Advisory Board) comprising five adult migrants with lived experience of accessing healthcare in the United Kingdom were also consulted at significant points over the course of the study.

## 1 INTRODUCTION

Vaccination is one of the world's most cost‐effective and successful public health interventions and is essential to reducing mortality and morbidity caused by serious infectious diseases. In the United Kingdom and Europe, several studies have suggested migrants are also an underimmunised group for routine vaccinations, with few systems in place to engage and catch up with older age groups.^1^, ^2^, ^3^, ^4^ Barriers include poor access despite availability, low confidence in vaccine safety and effectiveness, and low trust in public institutions and the wider health system.^2^, ^5^, ^6^, ^7^ Many of these same populations also suffered disproportionately worse health and economic outcomes because of the pandemic.^8^, ^9^ Faced with the COVID‐19 pandemic, scientists and governments rapidly set about developing and distributing safe and effective vaccines for COVID‐19 to help bring the pandemic under control and protect populations. However, the success of vaccine‐based protection measures hinges on high population uptake and coverage. Monitoring of the COVID‐19 vaccination roll‐out in high‐income countries revealed stark discrepancies in COVID‐19 vaccine uptake particularly affecting intersectionally marginalised populations, including migrants.^5^, ^10^, ^11^, ^12^, ^13^, ^14^, ^15^, ^16^

Health inequalities can be linked to wider social inequalities, including broader environmental, social and economic factors. Globally, COVID‐19 exacerbated inequalities experienced by some migrants and ethnically minoritised groups and highlighted the structural violence embedded within society.^17^, ^18^ Along with hostile immigration policies, institutional racism and xenophobia, the medical establishment has a long history of exploiting and mistreating black and some ethnically minoritised populations.^19^, ^20^ This is reflected in their poorer health outcomes compared to white groups. For example, rates of infant and maternal mortality, cardiovascular disease and diabetes are higher among Black and South Asian groups. The effects of this wider context on trust were also evident in widely reported conspiracy theories about population control and concerns of being used as ‘guinea pigs’ in the COVID‐19 vaccination drive, posing major barriers to vaccine uptake.^10^, ^21^, ^22^ Muddled and inconsistent messaging and a lack of leadership from Heads of State during acute phases of the pandemic also likely contributed to lower trust in the health system and allowed misinformation to thrive,^23^ particularly among migrant and ethnically minoritised groups. There were also clear information barriers for those with limited English language proficiency and the failure of governments to adequately adapt and disseminate essential messaging to diverse populations.^24^ Although governments later took steps to physically widen access to COVID‐19 vaccination for excluded groups,^25^, ^26^ these actions were not enough to repair their already eroded trust in public institutions and authorities. As we now begin to move from the pandemic to the endemic stages of COVID‐19, it is essential that we do not lose sight of the inequities highlighted or the momentum needed to tackle them. This is important not only to improve COVID‐19 vaccine equity but to improve the reach of routine vaccination programmes and improve health outcomes more broadly. The King's Fund recently stated that ‘a cross‐government strategy for reducing health inequalities and addressing the diverse health needs of all groups at risk of poor health and high mortality has never been more urgent’.^27^ This must be done sensitively, considering pre‐existing structures of oppression and mistrust and adequately accounting for populations' unique realities, lived experiences and diversity.

Various approaches based on behavioural insights theory have been used to increase the uptake of routine and other more established vaccinations. The World Health Organization's (WHO) Tailoring Immunisations Programme (TIP)^28^ employs the capability, opportunity, motivation and behaviour (COM‐B) model of behaviour change, the theoretical domains framework (TDF) and the behaviour change wheel (BCW)^29^, ^30^, ^31^ to understand and address vaccination behaviours. While TIP fosters in‐depth, mutual understanding among stakeholders recognises the complexity of vaccination behaviour and facilitates the implementation of interventions supporting change, it operates within a traditional research paradigm, where studies are designed and implemented by academics and research is done ‘on’ rather than ‘with’ communities. This approach may perpetuate inequities and hinder authentic participation, leading to under‐representation of these groups in research.^32^

In contrast, a participatory research paradigm directly considers power asymmetries and histories of oppression, gives value to the subjectivity of lived experience and actively involves individuals affected by the issue being studied as equal partners in the research process. Participatory research leads to knowledge that is locally situated and context‐specific, which is important for generating workable solutions to existing problems.^33^ In addition to enhancing community empowerment, it is argued that engaging communities in this way can advance the rigour, relevance and reach of research.^34^ To date, there have been shortcomings in the meaningful involvement of migrants in health research,^35^ which we see as an opportunity for improvement. The resurgence of interest in participatory research offers an opportunity to rethink approaches for addressing vaccine inequities and involving migrant populations in research. Adopting an inclusive, collaborative and community‐centred approach may advance efforts to close the global immunisation gap.

We therefore constructed this community‐based participatory research (CBPR) study with Congolese migrants in the United Kingdom to understand the complex mechanisms influencing their COVID‐19 vaccination attitudes, beliefs and behaviours, and use behavioural theory and participatory co‐design methods to translate these findings into a tailored intervention to strengthen their COVID‐19 vaccine uptake.

## 2 METHODS

### 2.1 Study aim, design and setting

This CBPR study aimed to co‐design a culturally tailored behaviour change intervention with Congolese migrants (non‐UK born) to strengthen their COVID‐19 vaccine uptake. It was conducted by a community‐academic coalition (including Congolese migrants, community, and academic stakeholders) from November 2021 to November 2022 in Hackney, United Kingdom, a diverse London borough. Community days (involving peer‐led qualitative in‐depth interviews and interactive poster walls) and co‐design workshops were conducted with Congolese migrants (see Table 1) and the CBPR approach was evaluated through participant feedback. Further context about the study, population, sampling, recruitment and data collection methods are described in a published protocol.^36^ All study resources and expenses were paid for by grants awarded to the St George's research team. Participants were financially compensated for participation using vouchers (1‐h interviews—£20; 2‐h workshops—£40) and reimbursed in cash for travel costs. Nonacademic coalition members were paid for their time (according to rates set out by NIHR INVOLVE guidance^37^) and Hackney Congolese Women Support Group and Hackney Refugee and Migrant Forum received financial donations to support their running, in addition to nonfinancial contributions (e.g., skills‐based training).^36^ A community celebration and presentation of key findings was held in July 2022.

**Table 1:** Inclusion and exclusion criteria of study participants.

Inclusion criteria	Exclusion criteria
Born in the Democratic Republic of Congo (DRC). Aged 18 or above. Currently residing in the United Kingdom. Willing and able to give informed consent.	Not migrant as per earlier definition. Not born in the DRC. Below the age of 18. Temporarily in the United Kingdom for holidays, visiting friends/relatives or other reasons. Lacking the capacity to consent, as determined by the Mental Capacity Act framework.

### 2.2 Study costs

This study cost approximately £17,500 to conduct, not including academic staff time. This included £7000 on general project spend (coalition member payments and expenses, participant vouchers and expenses, venue hire, catering and entertainment for end‐of‐study celebration event, stationery and other materials, professional artist hire), £4500 in one–off donations to nonacademic partners and £6000 on translation and transcription costs, using a professional translator from the London Congolese community.

### 2.3 Intervention development procedure

**Figure 1:**
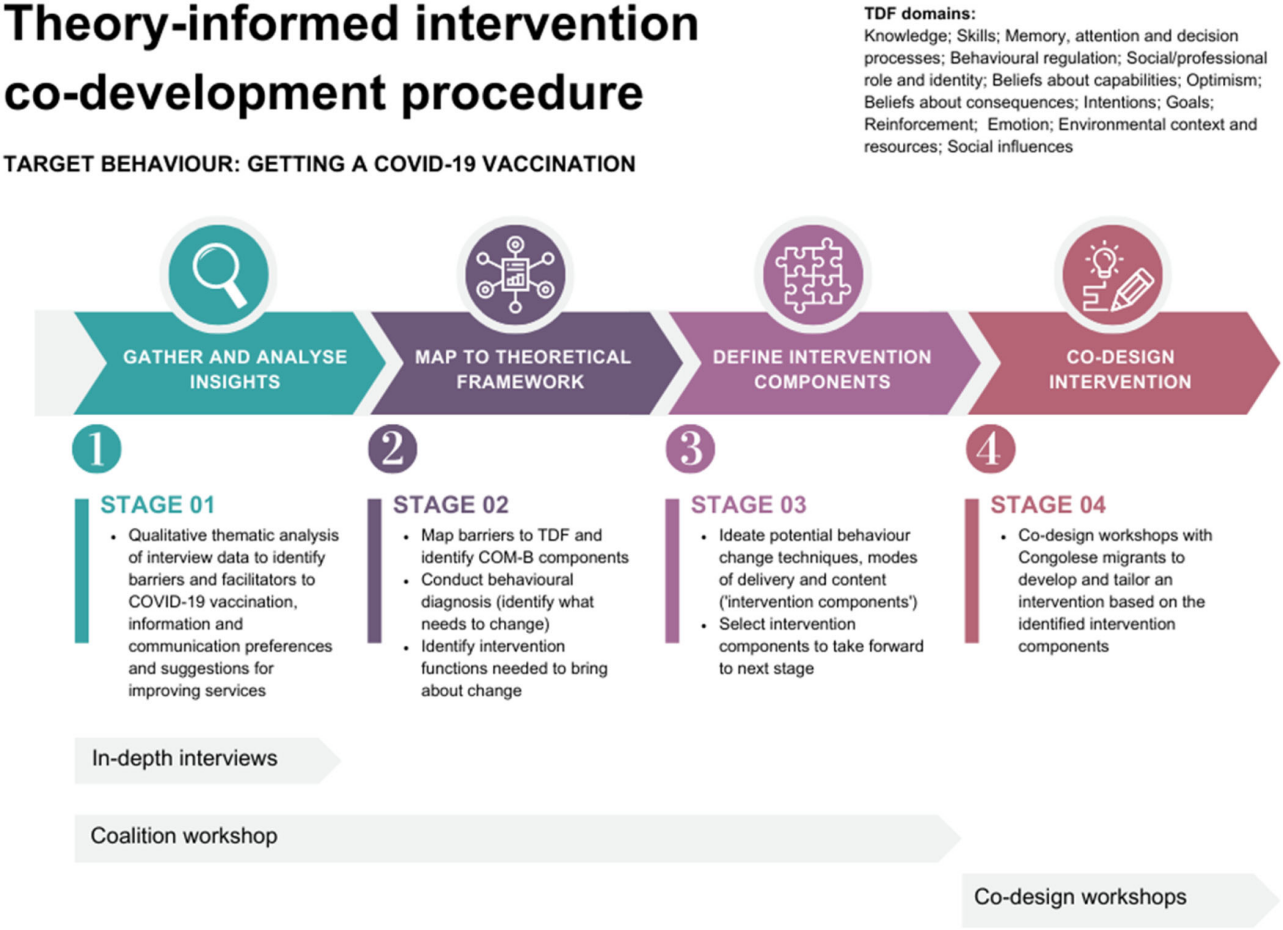
The four stages of the theory‐informed intervention co‐development procedure: gather and analyse insights; map to theoretical framework; define intervention components and co‐design intervention. The target behaviour was getting a COVID‐19 vaccination. COM‐B, capability, opportunity, motivation and behaviour; TDF, theoretical domains framework.

Michie et al.^29^ recommend several steps to design a behavioural change intervention, starting with defining the problem in behavioural terms and selecting a target behaviour the intervention should increase in the population. We defined our target behaviour as ‘getting a COVID‐19 vaccination’. The four stages of intervention development are outlined in Figure 1. First, data collected through in‐depth interviews and poster walls with Congolese migrants were thematically analysed^38^ collaboratively by the coalition to identify barriers and facilitators to vaccination, communication preferences, sociocultural values and suggestions for improving vaccination services. Barriers were mapped to the 14‐domain TDF,^30^, ^39^ COM‐B model and BCW^31^ and a behavioural diagnosis was made following Michie et al.,^29^ generating possible intervention functions (functions likely to be effective in achieving behaviour change) which represented a starting point for intervention development. The coalition brainstormed ideas for possible intervention components, reflecting on the qualitative findings and their specific sociocultural and local knowledge. ‘How Might We’ questions (a design thinking approach^40^) were used to aid creativity and problem‐solving. Three intervention components were agreed upon to take forward to co‐design workshops, which were felt to blend community desires with effective and contextually feasible approaches to change behaviour. These components were iterated on and refined by Congolese migrants during two, 2‐h co‐design workshops led by the Congolese coalition members (L. M. L., L. M. K., S. N.) with support from AFC and CH, resulting in a final, culturally tailored and co‐designed intervention. A local artist attended the workshops and recorded visual minutes.

## 3 RESULTS

Thirty‐two interviews and two co‐design workshops (*n* = 16, 8 per workshop) were conducted with Congolese migrants. Descriptive characteristics of the qualitative interview participants (*n* = 32) are shown in Table 2 and described briefly. Co‐design participants were drawn from this sample. Most (75%) of the interview participants were female, had a mean age of 52.6 years (SD: 11 years), and had lived in the UK for an average (mean) 14.3 years (SD: 7.5 years). Inclusion criteria were expanded to include two Congolese‐identifying but Angolan‐born participants, recognising the limitations of the original categories. Most participants spoke Lingala (88%) or French (63%); few spoke English (31%) and 47% considered themselves to have limited English proficiency (unable to read or write). All (100%) were registered with a general practitioner (GP). Interviewees were asked their COVID‐19 vaccination status and the number of doses received at the time of their interview (conducted from January to March 2022). Four (13%) answered ‘unvaccinated/0 doses’, 18 (56%) answered ‘1–2 doses’, 10 (31%) answered ‘3 or more doses’ and 1 (3%) answered ‘uncertain’. In the co‐design workshops, there was an almost even sex distribution (four women, four men in workshop 1; three women, five men in workshop 2).

**Table 2:** Characteristics of qualitative interview participants (*n* = 32).

Characteristic	*n* (%)
Migrant status	
Seeking asylum	6 (19%)
Refugee	13 (41%)
British (naturalised)	6 (19%)
Prefer not to say	5 (16%)
Other visa	2 (6%)
Age, years, mean (SD)	52.6 (11.0)
25–49	13 (41%)
50–64	15 (47%)
Over 65	4 (13%)
Gender	
Female	24 (75%)
Male	8 (25%)
Time since arrival in the United Kingdom (years), mean (SD)^a^	14.3 (7.5)
0–9	6 (19%)
10+	22 (69%)
20+	9 (28%)
Not available	2 (6%)
Country of birth	
Democratic Republic of Congo or Republic of Congo^b^	30 (94%)
Angola^c^	2 (6%)
Religion	
Christianity	32 (100%)
Marital status	
Single	18 (56%)
Married	10 (31%)
Other	4 (13%)
Currently have children <16 years of age living in the household	
Yes	15 (47%)
Languages spoken	
Lingala	28 (88%)
French	20 (63%)
English	10 (31%)
Other (Kikongo, Portuguese)	3 (9%)
Limited English proficiency (self‐reported, cannot read or write in English)	
Yes	15 (47%)
No	14 (44%)
No response	3 (9%)
Registered with GP	
Yes	32 (100%)
Given routine/childhood vaccination card in country of origin	
Yes	11 (34%)
No	17 (53%)
Don't know	4 (13%)
Brought routine/childhood vaccination card to the United Kingdom (*n* = 15 asked)	
Yes	4 (27%)
No	10 (67%)
Don't know	1 (7%)

Abbreviation: GP, general practitioner.

^a^ Where respondents answered the question ‘Time since arrived in the United Kingdom’ with ‘more than 10 years’, this was assigned the value of 10 years in the continuous distribution/mean calculation; ‘more than 20 years’ was assigned the value of 20 years; ‘more than 25 years’ was assigned the value of 25 years.

^b^ Countries were combined as many respondents answered ambiguously, that is, ‘Congo’.

^c^ We expanded our inclusion criteria to include two participants who were born in Angola but identified as Congolese.

### 3.1 Results part 1: Barriers and facilitators to COVID‐19 vaccination, information and communication preferences and values

Fourteen barrier concepts, organised under five topic headings (vaccine safety concerns, vaccine effectiveness concerns, vaccine necessity and norms, issues relating to information and communications and government distrust), and 10 facilitator concepts, organised under eight topic headings (accessibility of the vaccine, opportunity to discuss with a GP or other trusted source, higher risk perception and saliency of the disease, social influences, respect for authority, trust in government, belief in medical research process, desire to protect self and others) were identified (examples of data shown in Supporting Information: Table S1). Participants' information and communication preferences and cultural values were also identified.

#### 3.1.1 Barriers

Vaccine safety concerns included uncertainty about the COVID‐19 vaccine development process and speed, beliefs about consequences due to personal risk factors (e.g., blood clots), a negative experience (e.g., side effects from an earlier dose), knowledge of vaccine scares and historical events (e.g., contracting vaccine‐derived poliomyelitis) or belief in rumours and conspiracy theories about the vaccine's effects.

My issue was on the blood clot side because when I had my kid, I was bleeding a lot, I lost 1 litre plus. So, when I heard on the news that people were having blood clots I said, my God, it makes me feel really scared. (P5, female)

Yes, some children have become disabled after receiving polio vaccine. […] [They are afraid] because the side effects of vaccine have caused to their children to become disabled, and they don't want again to take the risk. (P2, female)

There were also concerns around the vaccine's effectiveness and the need for multiple doses or boosters. Participants questioned the necessity of the vaccine when it doesn't necessarily prevent infection and contrasted the COVID‐19 vaccine with other vaccines such as the influenza vaccine, which some perceived to be more effective. One participant said, “I prefer flu vaccine because that one will protect you” (P21, female).

Issues relating to information and communications were another important barrier. Many participants highlighted how language and literacy barriers had directly influenced their vaccination decisions, for example, not having access to an interpreter, or through exposure to misinformation and rumours in their social networks, causing fear and distress.

I refused [the vaccine] the first time… Because I came recently in the country, and I was not sick. I just came and I couldn't speak English. I refused. No, I wanted to have an interpreter to explain to me… (P28, female)

It was not easy for me [to get the vaccine] because there was so many rumours and I was questioned myself if do I have to take it or not. We came in this country to seek protection. (P4, female)

A few participants also said they felt confused and overwhelmed by the official information and public health messaging, which had been complicated and at times contradictory. For example,

I was scared and reluctant about the vaccines because I was confused with the information from research…. I was not sure because scientists were not clear in their language. (P6, female)

Widespread exposure to misinformation and rumours also made it difficult for participants to know what to believe and enhanced mistrust towards authorities and public institutions. Our data suggest that many participants felt the official public health communications used by the government and NHS were coercive, and this increased their scepticism of the response, including the vaccine. Many participants said they felt they were being ‘forced’ or ‘imposed’ to take the vaccine, that freedom of choice had been taken away, and this had made them question the government's motives behind the vaccination programme. For example,

I have been constantly receiving letter pushing me to receive vaccine. […] I would do it voluntarily but not by force. Now they are forcing people and I don't know what is hidden behind this vaccine? (P16, male)

Participants voiced concerns that they might be being exploited and used as ‘guinea pigs’ by the NHS and government and alluded to present‐day racism and historical events involving the exploitation of black and African populations by white Europeans. Some also commented that they felt bombarded by instructions and rules from the government and NHS about how to behave but these instructions lacked the information to help them feel safe or understand the rationale.

#### 3.1.2 Facilitators, information and communication preferences and values

Most participants knew how and where to get a COVID‐19 vaccine, suggesting that access was not a major barrier in this context. Facilitators to vaccination included having a dialogue with a GP or other trusted source (considered to be friends and family, local community organisations, teachers), social support to get vaccinated and seeing others from the community get vaccinated. Participants highlighted a preference for oral and visual communication, Lingala language, face‐to‐face, small group, and one‐to‐one dialogues. They highlighted several preferred information channels and meeting points, including barber shops, African food shops and restaurants, churches, parties, football/running clubs, local community support organisations and traditional and social media. Community, family, respecting elders, religion, and creative forms of expression (music, theatre, art, dance) were considered important. Participants expressed frustration that there had never been a workshop for their community. There was a strong demand for workshops, conversations and face‐to‐face meetings about COVID‐19, including explanation of risks, benefits of vaccination, transparent information about clinical trials, warnings about misinformation and what to expect after getting vaccinated.

### 3.2 Results part 2: Behavioural mapping exercise and selection of interventions

Most of the barrier data related to four TDF domains: beliefs about consequences, emotion, social influences and environmental context and resources, with smaller clusters of data related to optimism, decision‐making processes and deficits in knowledge (Supporting Information: Table S1). The mapping and behavioural diagnosis exercise^31^ identified that psychological capability (specifically: knowledge; decision processes), reflective motivation (intentions; beliefs about consequences; optimism), automatic motivation (emotions/fear) and social opportunity (social influences) needed to be addressed through the intervention design. Five (of nine) corresponding intervention functions were selected (for practical reasons) for the intervention development: education, persuasion, modelling, enablement and environmental restructuring (the relationships between these are shown in Supporting Information: Table S2). Possible intervention components (behaviour change techniques and mode of delivery) linked to these intervention functions that were generated by the coalition are summarised in Table 3.

**Table 3:** Intervention functions and potential behaviour change techniques, modes of delivery and types of content ideated during coalition workshop.

Intervention function	Behaviour change technique(s)	Ideas generated during the coalition workshop on how techniques could be applied to interventions and/or intervention content
Education	Providing information regarding behaviour/outcome	*Mode*(*s*): Workshops, public lectures, round tables and facilitated conversations (groups and one‐to‐one) led by GPs and other trusted messengers; adapt school curriculum; community members co‐design songs, dance, plays. *Content/details*: • Trusted messengers from local community organisation (HCWSG) and healthcare professionals deliver information about COVID‐19 vaccination, e.g., benefits of vaccination, risks and consequences of COVID‐19 infection (e.g., long COVID), debunking myths and conspiracy theories, information about COVID‐19 vaccine development. • More opportunities for patients to speak to GPs and healthcare professionals about vaccination informally (e.g., roundtables, townhalls). • Communities and community organisations involved in co‐designing hyper‐local messaging, delivered through creative and engaging formats (e.g., songs, dance, plays, posters). • COVID‐19 vaccination education and messaging built into the school/college curriculum (e.g., PSHE lessons, ESOL).
Enablement	Social support to do the behaviour/get vaccinated	*Mode(s)*: Peer support; community support groups; buddy systems; normalisation. *Content/details*: • Trusted community members/peers trained in discussing vaccination concerns, addressing uncertainties, providing and supporting access to official information, ‘show and tell’ of vaccination cards (peer support; normalisation). • Local community support groups established to help people make vaccination decisions (support groups). • Community encouraged to go with a partner or friend to a vaccination appointment (buddy system). • Long‐term campaigns about COVID‐19 vaccination (normalisation). • Information that highlights similarities of COVID‐19 vaccine and development process with other well‐known vaccines, e.g., flu vaccine (normalisation). • Adding COVID‐19 vaccination to routine health check‐ups in primary care (normalisation).
Environmental restructuring	1. Adding objects to the environment 2. Guidelines 3. Restructuring physical environment	*Mode(s)*: Adding tailored multimedia (posters, flyers, videos, etc.) to the local environment; guidelines/training manuals; grassroots funding and reorganisation; government accountability and action. *Content/details*: • Local community organisations to receive official health information which they can tailor to the local population and context (with funding and support). • Tailored vaccination information (e.g., posters, stickers, video clips) distributed in locally‐relevant places (physically and online), e.g., barber shops, Top Africa magazine, Facebook pages. • Guidelines/training manuals to support local community organisations in training peer supporters/role models. • Establish new funding streams and structures to support more grassroots and community‐centred approaches and information flow from communities to policymakers rather than top‐down instruction. • More accountability and action from the government in addressing people's fears and ensuring health equity, including providing transparent health/vaccination information and acknowledging past injustices to establish trust.
Modelling	Demonstration of the behaviour by others	*Mode(s)*: Community role models; demonstrations of getting vaccinated; ‘show and tell’ vaccination cards. *Content/details*: • Community role models trained to share key messages, facilitate conversations, show COVID‐19/routine vaccination cards, talk about their own vaccination experiences. • Friends, community members and role models to provide examples of the behaviour, so that people have something to aspire to, know what to expect, and have visual proof that it is safe, e.g., through plays, dance, songs, posters, pictures of local people getting vaccinated, campaigns, etc., which can be shared in local settings and on social media. • Use local people and ensure the right people are chosen by speaking to community organisations who know their populations – celebrities will evoke distrust in this community.
Persuasion	1. Credible source 2. Providing information 3. Feedback on behaviour 4. Feedback on outcome of behaviour 5. Salience of consequences 6. Persuasive communication 7. Positive framing	*Mode*(*s*): Trusted advocates, messengers and community role models; Community Champions; creative methods, e.g., plays, posters, pictures; local campaigns and hashtags; lectures, meetings and workshops led by healthcare professionals/experts. *Content/details*: • Trusted advocates and healthcare professionals/experts present feedback on positive outcomes of vaccination in the community/local area, e.g., number of safe vaccinations administered. • Healthcare professionals/experts present examples of negative health consequences that could occur as a result of not getting vaccinated, e.g., long COVID. • Trusted advocates and community members/role models share positive stories, testimonials and persuasive messages about why they got vaccinated, what to expect, and being a COVID‐19 Champion. • Peer‐led conversations using gentle encouragement, empathetic tone and positive framing of messages. • Communities co‐design local campaigns with culturally relevant and positive vaccination messages to share in the local area (e.g., in African food shops, barber shops, on public transport) and on social media (e.g., sharing a photo of yourself getting vaccinated, GIFs/stickers, hashtags, posters about being a COVID‐19 Champion) • Government‐led messages acknowledging past injustices against ethnic minority communities and long‐term efforts to rebuild trust.

Abbreviations: ESOL, English for speakers of other languages; PSHE, personal, social, health and economic.

Three broad intervention components were then selected by consensus. The first component was centred around workshops, as there was a strong demand for this type of activity within the community. The second component focused on creative performance‐based activities like dance, songs and plays. The third component focused on visual media such as posters and GIFs. These components were chosen because the participants emphasised the significance of creative expression in their culture. Additionally, they expressed a preference for visual and oral forms of communication.

### 3.3 Results part 3: Outputs of co‐design workshops

Participants customised and tailored the intervention components in the co‐design workshops, resulting in a final intervention comprising community‐led workshops, plays and posters. Table 4 shows how the intervention components addressed the intervention functions identified in the behavioural diagnosis.

**Table 4:** Table summarising how intervention components addressed corresponding intervention functions identified in the behavioural diagnosis.

Intervention component and means of addressing the function	Intervention function				
	Education	Environmental restructuring	Persuasion	Modelling	Enablement
*Workshops*	**✓✓**	**✓✓**	**✓✓**	**✓**	**✓**
Means of addressing the function	Provided information regarding the behaviour/outcome (COVID‐19 vaccination) and opportunity for dialogue around COVID‐19 vaccination	Added a previously unavailable service to the local environment in a desired format (e.g., oral communication, dialogue‐based)	Used credible sources to enhance public trust (engagement of experts with the community, valuing cultural and experiential knowledge, inclusion of trusted community leaders); positive framing and persuasive communication around COVID‐19 vaccination; provided information and feedback on behaviour/outcome	Community leaders shared positive experiences of vaccination	Provided access to peer, community and professional support to do the behaviour and addressed uncertainties around vaccination through dialogue
*Plays*	**✓**	**✓**	**✓✓**	**✓✓**	**✓**
Means of addressing the function	Provided information regarding vaccination; positive framing and tailoring of messages; used preferred visual and storytelling formats	Introduced a new means of engaging the community around health and vaccination	Scripts used empathy and showed understanding of the community's concerns; messages were framed and delivered in a way the audience understood and related to (e.g., cultural/local references, relatable characters, humour)	Actors modelled desired vaccination behaviour; relatable and representative characters	Storylines normalised COVID‐19 vaccination
*Posters*	**✓**	**✓**	**✓✓**	**✓**	**✓**
Means of addressing the function	Provided information regarding vaccination	Added tailored multimedia to the local environment	Positive framing and persuasive communication; vaccination messages appealed to values of community, protection, agency and liberty; culturally relevant imagery and colours	Used pictures of known community members to model and encourage vaccination behaviour	Signposted to community organisations, workshops, information and support to do the behaviour

*Note*: **✓✓** dominant function; **✓** auxiliary function.

#### 3.3.1 Intervention component 1: Community‐led workshops

Both participant groups co‐designed a community‐led workshop plan (Supporting Information: Table S3). Key tailoring needs included local, in‐person meetings, Lingala language (with interpreters if possible) and regular, scheduled sessions (favoured over pop‐ups for dependability and frequency) on Friday and Saturday. Participants preferred for workshops to be delivered by the local community organisation (HCWSG) with specialists and health professionals as speakers. They highlighted a desire for two‐way communication, with opportunities to ask questions and discuss experiences. There was a demand for covering wider health topics in addition to COVID‐19 vaccination information.

#### 3.3.2 Intervention component 2: Short plays

**Figure 2:**
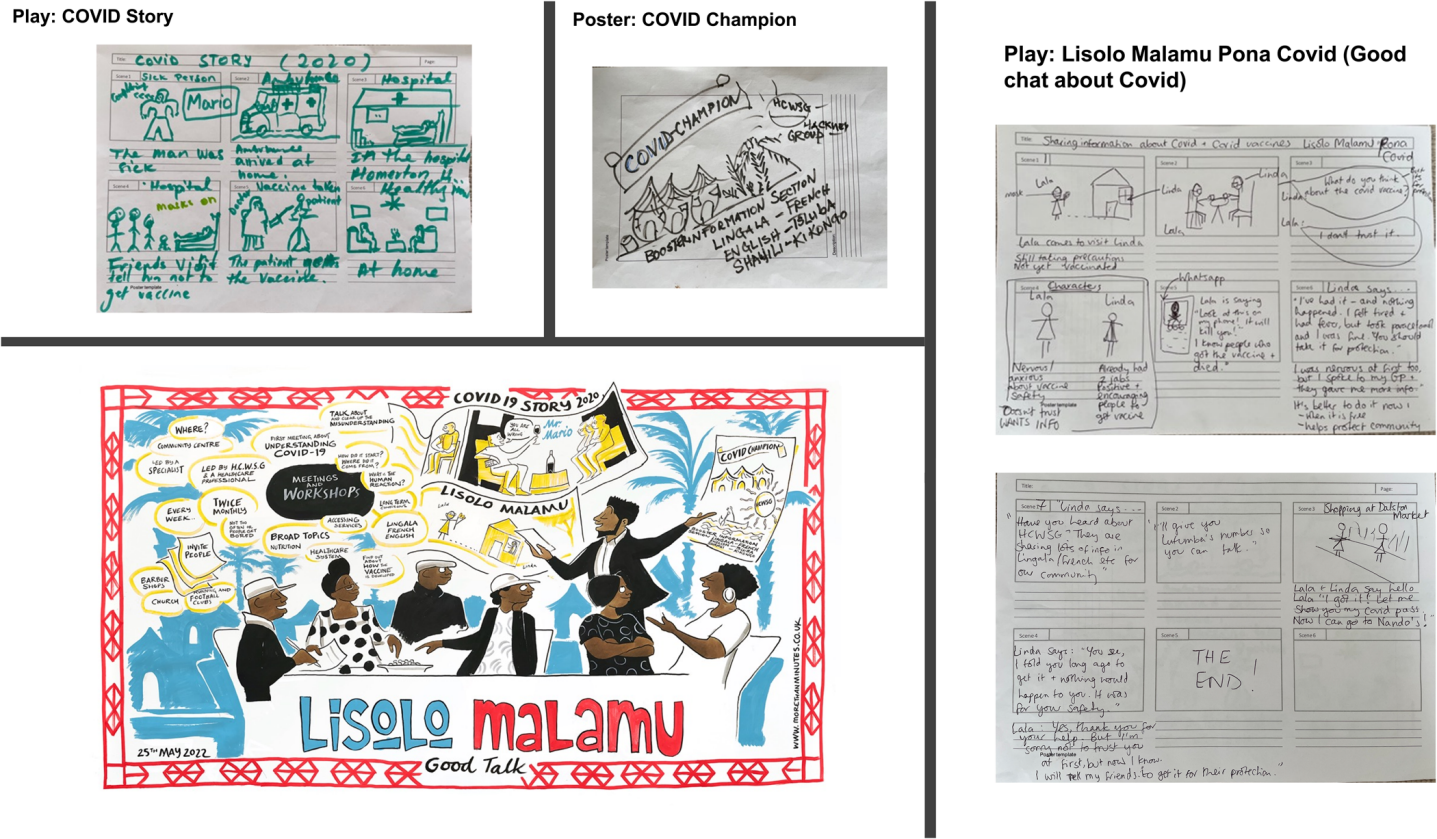
Examples of the co‐designed plays and posters for intervention components 2 and 3. Bottom left: The artist's live drawing of the workshops and final intervention comprising workshops, plays and posters.

Participants co‐designed short plays (Figure 2) using storyboards. Plays utilised storytelling to highlight common barriers, concerns and fears about vaccination in the community identified during the interviews and used culturally adapted behaviour change techniques, such as modelling positive vaccination experiences/behaviour and positively framing messages through relatable characters, local settings, cultural references, customs and humour to encourage vaccine uptake.

#### 3.3.3 Intervention component 3: Posters and flyers

Participants co‐designed campaign‐like posters about COVID‐19 vaccination and invitational flyers for the workshops (Figure 2). They preferred to use rich, eye‐catching colours (by contrast, black was felt to signify death), culturally relevant imagery (e.g., Congolese scenery, ways of life), photos of local people to convey credibility and Lingala language. They wanted printed and digital versions to share through a range of channels.

#### 3.3.4 Artist's impression of workshops and intervention components (visual minutes)

The visual minutes from the co‐design workshops (Figure 2) have been reproduced to support funding applications and share the study findings and process with a range of stakeholders.

### 3.4 Feedback on the participatory process

We received 38 completed feedback forms from the interviews and co‐design workshops. Feedback was positive: participants said they felt welcomed and valued in the community, could express opinions honestly, and found the discussion important. They said they found the workshops fun and enjoyed the participatory and sociable nature.

### 3.5 Dissemination

The findings and intervention were shared through a community celebration event in July 2022, attended by 45 Congolese community members and participants, a local councillor, and live streamed by an African YouTube channel with 18,000 subscribers. Academics and policymakers were informed at two international conferences. A project brief will be shared with local and national stakeholders.

## 4 DISCUSSION

This study describes how co‐design and CBPR approaches were used to develop a culturally tailored behaviour change intervention to strengthen COVID‐19 vaccination uptake in a Congolese migrant population in the United Kingdom. Congolese migrants were found to experience similar barriers to COVID‐19 vaccination as identified in other migrant and ethnic minority groups. Participants indicated a preference for oral and visual communications and receiving vaccination information via a trusted intermediary. They were also keen for interventions to reflect their Congolese customs and heritage. These barriers and preferences were addressed through co‐designed workshops, plays and posters. This study effectively demonstrates how behavioural theory can be adapted to a participatory approach to co‐design a vaccination intervention.

The key barriers to COVID‐19 vaccination identified in our study population were concerns about the vaccine's safety, effectiveness, and side effects, information and communication issues (such as language barriers, exposure to misinformation, inadequate or confusing official messaging) and general mistrust of the COVID‐19 vaccination programme. Concerns about the vaccine were mostly attributed to its novelty and perceived insufficient testing time. Similar barriers have been reported among other migrant populations,^10^, ^23^, ^41^, ^42^, ^43^, ^44^, ^45^ suggesting a need for a more nuanced and responsive approach that addresses the specific concerns and worldviews of diverse communities and builds trust. A key aspect of fostering vaccine acceptance lies in enhancing institutional and interpersonal trust and trust in vaccines,^46^, ^47^ which may be achieved by actively listening to the concerns of various groups and prioritising transparent and clear communication, especially during emergencies. Surprisingly, access to vaccines was not a major barrier in our study, suggesting that government efforts to widen access to vaccination for marginalised groups during the pandemic were largely successful. Nevertheless, the limited impact of these efforts on increasing uptake in this population due to other prevailing barriers emphasises the need for contextually‐tailored initiatives, rather than a one‐size‐fits‐all approach.

Participants indicated a preference for visual, oral, dialogue‐based and face‐to‐face forms of communication and put trust in healthcare professionals and community leaders and members. Interestingly, despite all participants being registered with a GP, they still reported barriers to vaccine uptake. This suggests that contact with a healthcare professional alone may not be sufficient to facilitate uptake and indicates potential shortcomings in primary care services’ provision of culturally competent care for this population. These findings underscore the urgent need for interventions and service adaptations that better cater to the linguistic needs and cultural diversity of migrant populations. The critical role of community connectors in facilitating vaccination opportunities must also be recognised and integrated into intervention strategies.

Participants emphasised a sense of pride in their heritage, customs and community, and wanted to design interventions that reflected their cultural identity. They specifically highlighted the significance of storytelling, rich colours, and illustrations depicting their homeland. Identifying and incorporating these cultural elements into interventions may ensure they are more representative and relatable to the target population. Such culturally sensitive approaches may help to effectively engage with marginalised groups and foster a sense of belonging and inclusion, which has been shown to influence the health decision‐making process.^48^ These unique findings emphasise the value of actively involving communities in co‐designing and tailoring interventions. A participatory approach not only ensures interventions are culturally appropriate but also promotes a sense of ownership and investment within the community, which may enhance interventions' effectiveness and impact.

Previous literature highlights gaps in understanding around how to develop tailored and targeted health interventions involving migrants, beyond engaging with community‐based organisations and using culturally appropriate messaging.^49^ There are also limited examples of participatory, co‐designed vaccination interventions involving migrant populations.^50^, ^51^, ^52^, ^53^, ^54^, ^55^ Our study addresses this gap and offers a valuable example of a community‐engaged approach to co‐designing a vaccination intervention for an underserved migrant community. Our study builds on previous work which used WHO TIP methodology to develop a tailored intervention to increase vaccine acceptance in a Somali community,^56^ by showing how behavioural theory can be employed in a participatory study design. It also complements a study in New Zealand that used behavioural theory and cultural insights to co‐design a lifestyle support mobile health intervention with Maori/Pasifika populations.^57^ A strength of the New Zealand study was its use of ethnic‐specific models of health alongside the TDF, representing the worldviews of Maori and Pasifika populations. Future studies seeking to use behavioural theory in the development of interventions with migrant populations could explore developing migrant‐specific models of health with communities, as a means of ensuring culturally specific beliefs, values and worldviews are more robustly translated into behaviour change techniques while equally valuing Western and migrant worldviews.

Our findings align with other research which highlights preferences for face‐to‐face^58^, ^59^ and oral communication^60^, ^61^ among migrant populations, as well as strategies that build or reinforce trust.^59^, ^62^, ^63^ They also align with systematic reviews that have indicated that culturally adapted interventions may be effective in community settings.^64^, ^65^ However, our use of sociocultural elements and community members to facilitate engagement with the intervention went beyond the surface‐level cultural adaptations common to behavioural interventions, such as language translation or reading level adjustments,^65^ representing an advancement on current literature. Other studies have indicated limitations in culturally competent care for refugees and migrants,^66^, ^67^ which our findings allude to, including the need for greater refugee participation and perspectives in the practice of cultural competence and recognition of structural barriers.^68^, ^69^ This emphasises the need for a whole‐system approach to creating a more enabling environment to facilitate vaccine uptake. Future interventions may be strengthened by incorporating multi‐level intervention components and identifying policy categories that support their delivery.

A key strength of our study was its community‐centred, participatory approach. Participatory research aims to reinforce local capacity and solutions and promote transformative change.^34^, ^69^, ^70^ However, existing participatory health research with migrants has been criticised for inadequately including migrants in developing health interventions.^35^ In our study, we enhanced community capacity through a partnership approach that shared power, recognised and celebrated community assets and expertise and provided skills‐based training and leadership opportunities for community partners. Several studies involving marginalised populations have demonstrated the benefits of involving community members as health promoters or advocates to build trust and facilitate the uptake of interventions,^59^, ^62^, ^63^ including targeted initiatives to increase COVID‐19 vaccine uptake with refugees and migrants^52^ and COVID‐19 ‘Community Champions’ schemes implemented in local authorities.^71^ However, our study went beyond these, by actively involving community members in designing and leading the study. Our Congolese partners played a vital role in building relationships and establishing trust, providing valuable cultural and experiential knowledge to tailor activities and ensure participants felt valued and heard. This was reflected in the high attendance of participants in research and dissemination activities and overwhelmingly positive feedback received during the evaluation. Our focus on community assets and the resourcefulness of underserved communities like migrants challenges deficit models which often underpin behaviour change models and solely attribute barriers to language difficulties and issues related to access and trust. Using participatory methods, we demonstrated that underserved communities are resilient and can find real‐world solutions to their health needs.

Future studies and initiatives should build on this community‐centred, participatory approach. Collaborative partnerships with people and communities are now considered critical in healthcare, and recent legislation in England^72^ aims to address health inequalities highlighted by the pandemic and provide more tailored care to diverse communities. However, there is still relatively limited guidance on how to do this well in research or practice. We provided details of our budget and participatory process for transparency and to highlight challenges and costs for others working in this space. While the increased attention on collaborative approaches is positive, funders, authorities and researchers must be cognisant of how inherent biases and systemic racism may serve to widen inequalities despite their good intentions and proactively address this. For instance, they should be sensitive to how their actions to address inequity may be perceived and how they may inadvertently heighten the sense of exclusion felt by other underserved groups, particularly migrants. It will be crucial to recognise and support migrant communities and smaller organisations that informally support their communities by creating accessible local funding and capacity‐building mechanisms. Our study funded a black‐led organisation to lead community‐based research addressing issues important to their community and provided personal development opportunities to build community capacity. However, an alarmingly low number of black‐led organisations were awarded funding in the COVID‐19 response and in the community and voluntary sector in general.^73^, ^74^, ^75^, ^76^, ^77^ As such, our study contributes to understanding how community engagement and participatory research can promote equity in migrant health and help dismantle power structures hindering vaccine uptake and perpetuating harm among these communities.

### 4.1 Limitations

Our study's primary limitation is the lack of implementation or evaluation of the intervention due to time and budget constraints. As a result, we cannot draw conclusions on the intervention's feasibility, effectiveness, or acceptability. However, we are pleased to report that our community partner has successfully obtained further fundraising and capacity‐building support locally to enable them to continue building on this work. Challenges of conducting participatory research in the current academic funding environment have been noted.^69^, ^78^, ^79^ Our study underscores the need to restructure research funding to better accommodate the unique requirements of participatory, community‐based research, including longer timelines and the resource‐intensive nature of forming community partnerships and long‐term engagement.

Despite our efforts to foster full participation, power imbalances still existed in our approach. The study was initiated by academics who had secured funding for research on improving vaccine uptake among migrant communities. The onset of the pandemic made addressing COVID‐19 vaccination barriers a pressing concern among migrant communities, aligning our research topic with community needs and facilitating our partnership. However, it may be more challenging to justify co‐designing community‐based interventions to strengthen routine vaccine uptake if communities do not consider this a research priority. The idea for the behavioural underpinning of the intervention was also put forward by the academic partner and led to an intervention predominantly focused on addressing individual modifiable behaviours. Consideration should be given as to whether the use of this framework may have limited the impact of the participatory approach or impeded engagement with upstream factors such as systemic racism and discrimination, which are recognised to influence ethnic inequities in vaccine hesitancy.^80^ Streuli et al., for example, raised concerns about their use of design thinking and neo‐liberal ideologies in designing a vaccination education intervention for Somali refugees, and their potential impact on reinforcing structural inequalities.^81^ Future research should aim to identify the most effective ways of conducting participatory research with communities, being sensitive to their unique needs and context while also addressing broader systemic factors influencing vaccine hesitancy. Evaluating our intervention could help quantify and clarify the relative benefits of a community‐engaged and behaviourally informed approach.

## 5 CONCLUSIONS AND NEXT STEPS

The worse health outcomes of adult migrant populations during the COVID‐19 pandemic and their widely reported barriers to COVID‐19 vaccination have demanded exploration into more tailored interventions to increase vaccine uptake, which consider local context, including personal histories, power dynamics, preferences and needs and are developed and implemented in close collaboration with the target population. They have also highlighted wider inequalities and prompted research into ways of better engaging underserved adult groups specifically in vaccination campaigns, learnings from which can be adapted and used for strengthening routine immunisation programmes. This study reports on the theory‐informed co‐design of a tailored COVID‐19 vaccination intervention to address these complex challenges in an underserved Congolese migrant population in London. It provides an example of how interventions can be informed by behavioural theory and co‐designed with communities, ensuring cultural insights, values and preferences are incorporated. Our participatory approach^36^ offers one possible model for engaging with underserved communities in an empowering and equitable way, demonstrating how academic and community partners can better foster mutual exchange of expertise and work effectively together outside of traditional power structures. The next steps will involve refining, implementing and testing the intervention, and potentially adapting and expanding the content to routine vaccinations and wider health needs, as requested by study participants and to address gaps exacerbated by the pandemic. The findings also hold relevance to the co‐development and implementation of other health interventions and health promotion activities with migrants and other similar communities. Future research should build on this empowering approach to engaging with underserved migrant communities, with the goal of developing, implementing and evaluating more sensitive vaccination services and interventions which respond to migrant communities' unique needs and realities. Restructuring research funding to better accommodate the requirements of participatory, community‐based research will be needed to support such initiatives and promote equitable healthcare for marginalised populations.

## AUTHOR CONTRIBUTIONS

Alison F. Crawshaw had the initial idea for this research study. Alison F. Crawshaw, Laura M. Lutumba, Sarah Nkembi, Lusau M. Kitoko and Caroline Hickey jointly conceptualised and conducted the study, including investigation, recruitment, analysis and project administration. Alison F. Crawshaw trained the study team as peer researchers, managed the study and budget and wrote the original draft. Sally Hargreaves and Alice S. Forster provided supervisory support and advice during the study. All authors supported the development and refinement of study tools and procedures. All authors read and approved the final manuscript.

## CONFLICT OF INTEREST STATEMENT

The authors declare no conflict of interest.

## ETHICS STATEMENT

Ethics was granted by the St George's, University of London Research Ethics Committee (REC reference 2021.0128). All participants provided informed consent and were older than 18 years at the time of recruitment to the study.

## Supporting information

Supporting information.

## Data Availability

The data that support the findings of this study are available from the corresponding author upon reasonable request.

## References

[1] Carter J , Mehrotra A , Knights F , et al. “We don't routinely check vaccination background in adults”: a national qualitative study of barriers and facilitators to vaccine delivery and uptake in adult migrants through UK primary care. BMJ Open. 2022;12(10):e062894. 10.1136/bmjopen-2022-062894 PMC9557795 36216433

[2] Crawshaw AF , Farah Y , Deal A , et al. Defining the determinants of vaccine uptake and undervaccination in migrant populations in Europe to improve routine and COVID‐19 vaccine uptake: a systematic review. Lancet Infect Dis. 2022;22(9):e254‐e266. 35429463 10.1016/S1473-3099(22)00066-4 PMC9007555

[3] WHO . Ensuring the integration of refugees and migrants in immunization policies, planning and service delivery globally. In world health organization ed. Global Evidence Review on Health and Migration (GEHM) Series. WHO; 2022:21‐33. 35981261

[4] Deal A , Halliday R , Crawshaw AF , et al. Migration and outbreaks of vaccine‐preventable disease in Europe: a systematic review. Lancet Infect Dis. 2021;21(12):e387‐e398. 34626552 10.1016/S1473-3099(21)00193-6

[5] ECDC . Reducing COVID‐19 transmission and strengthening vaccine uptake among migrant populations in the EU/EEA: Technical Report. European Centre for Disease Prevention and Control; 2021.

[6] World Health Organization . Strengthening COVID‐19 Vaccine Demand and Uptake In Refugees and Migrants: An Operational Guide to Support All Those Responsible for Planning and Implementing the Rollout of Covid‐19 Vaccine to Refugees and Migrants at National and Local Levels, 14 March 2022. World Health Organization; 2022. nCoV/immunization/demand_planning/refugees_and_migrants/2022.1

[7] WHO . Strengthening COVID‐19 Vaccine Demand and Uptake in Refugees and Migrants: An Operational Guide. World Health Organization Headquarters; 2022.

[8] Hayward SE , Deal A , Cheng C , et al. Clinical outcomes and risk factors for COVID‐19 among migrant populations in high‐income countries: a systematic review. J Migr Health. 2021;3:100041. 33903857 10.1016/j.jmh.2021.100041 PMC8061095

[9] Mackey K , Ayers CK , Kondo KK , et al. Racial and ethnic disparities in COVID‐19‐Related infections, hospitalizations, and deaths: a systematic review. Ann Intern Med. 2021;174(3):362‐373. 33253040 10.7326/M20-6306 PMC7772883

[10] Deal A , Hayward SE , Huda M , et al. Strategies and action points to ensure equitable uptake of COVID‐19 vaccinations: a national qualitative interview study to explore the views of undocumented migrants, asylum seekers, and refugees. J Migr Health. 2021;4:100050. 34075367 10.1016/j.jmh.2021.100050 PMC8154190

[11] European Centre for Disease Prevention and Control . *Reducing COVID‐19 Transmission and Strengthening Vaccine Uptake Among Migrant Populations in the EU/EEA—3 June 2021*. ECDC; 2021.

[12] Hargreaves S , Hayward SE , Noori T , McKee M , Kumar B . COVID‐19: counting migrants in. Lancet. 2021;398(10296):211‐212. 10.1016/S0140-6736(21)01339-8 PMC8285119 34274061

[13] Dolby T , Finning K , Baker A , et al. Monitoring sociodemographic inequality in COVID‐19 vaccination uptake in England: a national linked data study. J Epidemiol Community Health. 2022;76(7):646‐652. 35470259 10.1136/jech-2021-218415

[14] Harris OO , Perry TE , Johnson JK , et al. Understanding the concept of trust and other factors related to COVID‐19 vaccine intentions among Black/African American older adults prior to vaccine development. SSM Qual Res Health. 2023;3:100230. 36785539 10.1016/j.ssmqr.2023.100230 PMC9898052

[15] Razai MS , Osama T , McKechnie DGJ , Majeed A . Covid‐19 vaccine hesitancy among ethnic minority groups. BMJ. 2021;372:n513. 33637577 10.1136/bmj.n513

[16] Nguyen LH , Joshi AD , Drew DA , et al. Self‐reported COVID‐19 vaccine hesitancy and uptake among participants from different racial and ethnic groups in the United States and United Kingdom. Nat Commun. 2022;13(1):636. 35105869 10.1038/s41467-022-28200-3 PMC8807721

[17] Burgess RA , Osborne RH , Yongabi KA , et al. The COVID‐19 vaccines rush: participatory community engagement matters more than ever. Lancet. 2021;397(10268):8‐10. 33308484 10.1016/S0140-6736(20)32642-8 PMC7832461

[18] McClure ES , Vasudevan P , Bailey Z , Patel S , Robinson WR . Racial capitalism within public health—how occupational settings drive COVID‐19 disparities. Am J Epidemiol. 2020;189(11):1244‐1253. 32619007 10.1093/aje/kwaa126 PMC7337680

[19] Kennedy BR , Mathis CC , Woods AK . African Americans and their distrust of the health care system: healthcare for diverse populations. J Cult Divers. 2007;14(2):56‐60. 19175244

[20] Bailey ZD , Krieger N , Agénor M , Graves J , Linos N , Bassett MT . Structural racism and health inequities in the USA: evidence and interventions. Lancet. 2017;389(10077):1453‐1463. 28402827 10.1016/S0140-6736(17)30569-X

[21] Knights F , Carter J , Deal A , et al. Impact of COVID‐19 on migrants' access to primary care and implications for vaccine roll‐out: a national qualitative study. Br J Gen Pract. 2021;71(709):e583‐e595. 33875420 10.3399/BJGP.2021.0028 PMC8216266

[22] Vandrevala T , Hendy J , Hanson K , Alidu L , Ala A . Unpacking COVID‐19 and conspiracy theories in the UK Black Community. Br J Health Psychol. 2022;28:482‐498. 36397650 10.1111/bjhp.12636 PMC11497291

[23] Crawshaw AF , Deal A , Rustage K , et al. What must be done to tackle vaccine hesitancy and barriers to COVID‐19 vaccination in migrants? J Travel Med. 2021;28(4):taab048. 33772312 10.1093/jtm/taab048 PMC8083646

[24] Nezafat Maldonado BM , Collins J , Blundell HJ , Singh L . Engaging the vulnerable: a rapid review of public health communication aimed at migrants during the COVID‐19 pandemic in Europe. J Migr Health. 2020;1:100004. 33447830 10.1016/j.jmh.2020.100004 PMC7661962

[25] European Centre for Disease Prevention and Control (ECDC) . *COVID‐19 Vaccination and Prioritisation Strategies in the EU/EEA*. ECDC; 2020.

[26] European Centre for Disease Prevention and Control (ECDC) *. Key Aspects Regarding the Introduction and Prioritisation of COVID‐19 Vaccination in the EU/EEA and the UK*. ECDC; 2020.

[27] The King's Fund . The health of people from ethnic minority groups in England Online. 2021. Accessed July 1, 2021. https://www.kingsfund.org.uk/publications/health-people-ethnic-minority-groups-england

[28] WHO . *Tailoring immunization programmes (TIP). World Health Organization Regional Office for Europe (WHO EURO)*. 2023. https://www.who.int/europe/activities/tailoring-immunization-programmes-(tip)

[29] Michie S , Atkins L , West R . The Behaviour Change Wheel: A Guide to Designing Interventions. Vol 1003, 1st ed. Silverback Publishing; 2014:1010.

[30] Cane J , O'Connor D , Michie S . Validation of the theoretical domains framework for use in behaviour change and implementation research. Implementation Science. 2012;7(1):37. 22530986 10.1186/1748-5908-7-37 PMC3483008

[31] Michie S , Atkins L , West R . The Behaviour Change Wheel: A Guide to Designing Interventions. Silverback Publishing; 2014. www.behaviourchangewheel.com

[32] Smirnoff M , Wilets I , Ragin DF , et al. A paradigm for understanding trust and mistrust in medical research: the Community VOICES study. AJOB Empirical Bioethics. 2018;9(1):39‐47. 29368998 10.1080/23294515.2018.1432718 PMC6092744

[33] World Health Organization . Participatory Health Research With Migrants: A Country Implementation Guide. World Health Organization, Regional Office for Europe; 2022.

[34] Balazs CL , Morello‐Frosch R . The three R's: how community based participatory research strengthens the rigor, relevance and reach of science. Environ Justice. 2013;6(1):9‐16. 10.1089/env.2012.0017 PMC3832061 24260590

[35] Rustage K , Crawshaw A , Majeed‐Hajaj S , et al. Participatory approaches in the development of health interventions for migrants: a systematic review. BMJ Open. 2021;11(10):e053678. 10.1136/bmjopen-2021-053678 PMC8548676 34697122

[36] Crawshaw AF , Hickey C , Lutumba LM , et al. Codesigning an intervention to strengthen COVID‐19 vaccine uptake in Congolese migrants in the UK (LISOLO MALAMU): a participatory qualitative study protocol. BMJ Open. 2023;13(1):e063462. 10.1136/bmjopen-2022-063462 PMC9842599 36639215

[37] NIHR . Payment guidance for members of the public considering involvement in research. 2021. Accessed December 1, 2021. https://www.nihr.ac.uk/documents/payment-guidance-for-members-of-the-public-considering-involvement-in-research/27372

[38] Braun V , Clarke V . Using thematic analysis in psychology. Qual Res Psychol. 2006;3(2):77‐101.

[39] Michie S . Making psychological theory useful for implementing evidence based practice: a consensus approach. Qual Saf Health Care. 2005;14(1):26‐33. 15692000 10.1136/qshc.2004.011155 PMC1743963

[40] IDEO.org . Design Thinking Design Kit: how might we. Accessed June 1, 2021. https://www.designkit.org/methods/3

[41] Abba‐Aji M , Stuckler D , Galea S , McKee M . Ethnic/racial minorities' and migrants' access to COVID‐19 vaccines: a systematic review of barriers and facilitators. J Migr Health. 2022;5:100086. 35194589 10.1016/j.jmh.2022.100086 PMC8855618

[42] Magee L , Knights F , McKechnie DGJ , Al‐bedaery R , Razai MS . Facilitators and barriers to COVID‐19 vaccination uptake among ethnic minorities: a qualitative study in primary care. PLoS One. 2022;17(7):e0270504. 35802738 10.1371/journal.pone.0270504 PMC9269906

[43] Mahimbo A , Kang M , Sestakova L , Smith M , Dawson A . Factors influencing refugees’ willingness to accept COVID‐19 vaccines in Greater Sydney: a qualitative study. Aust N Z J Public Health. 2022;46(4):502‐510. 35555951 10.1111/1753-6405.13252 PMC9347689

[44] Liddell BJ , Murphy S , Mau V , et al. Factors associated with COVID‐19 vaccine hesitancy amongst refugees in Australia. Eur J Psychotraumatol. 2021;12(1):1997173. 34868488 10.1080/20008198.2021.1997173 PMC8635584

[45] Shaw J , Anderson KB , Fabi RE , et al. COVID‐19 vaccination intention and behavior in a large, diverse, U.S. refugee population. Vaccine. 2022;40(9):1231‐1237. 35125223 10.1016/j.vaccine.2022.01.057 PMC8806127

[46] Adhikari B , Yeong Cheah P , von Seidlein L . Trust is the common denominator for COVID‐19 vaccine acceptance: a literature review. Vaccine X. 2022;12:100213. 36217424 10.1016/j.jvacx.2022.100213 PMC9536059

[47] Bollyky TJ , Hulland EN , Barber RM , et al. Pandemic preparedness and COVID‐19: an exploratory analysis of infection and fatality rates, and contextual factors associated with preparedness in 177 countries, from Jan 1, 2020, to Sept 30, 2021. Lancet. 2022;399(10334):1489‐1512. 35120592 10.1016/S0140-6736(22)00172-6 PMC8806194

[48] Crawshaw AF , Vandrevala T , Knights F , et al. Navigating COVID‐19 vaccination choices: The intersecting dynamics of institutional trust, belonging and message perception among Congolese migrants in the UK (A reflexive thematic analysis). 2023. 10.1371/journal.pgph.0002620 PMC11236099 38985733

[49] Laverack G . ‘Leaving no one behind’: the challenge of reaching migrant populations. Challenges. 2018;9(2):37.

[50] Hui C , Dunn J , Morton R , et al. Interventions to improve vaccination uptake and cost effectiveness of vaccination strategies in newly arrived migrants in the EU/EEA: a systematic review. Int J Environ Res Public Health. 2018;15(10):2065. 30241320 10.3390/ijerph15102065 PMC6210200

[51] Streuli S , Ibrahim N , Mohamed A , et al. Development of a culturally and linguistically sensitive virtual reality educational platform to improve vaccine acceptance within a refugee population: the SHIFA community engagement‐public health innovation programme. BMJ Open. 2021;11(9):e051184. 10.1136/bmjopen-2021-051184 PMC8442061 34521673

[52] Berrou I , Hamilton K , Cook C , et al. Leaving no one behind: interventions and outcomes of the COVID‐19 vaccine maximising uptake programme. Vaccines. 2022;10(6):840. 35746447 10.3390/vaccines10060840 PMC9227842

[53] Davis H , Elmer S , Graves K , Learmonth C . Codesign and community outreach to create COVID‐19 safe communities: a Karen community case study. Front Public Health. 2023;11:1081767. 37033045 10.3389/fpubh.2023.1081767 PMC10079966

[54] Fisher H , Chantler T , Denford S , et al. Development of a multicomponent intervention to increase parental vaccine confidence and young people's access to the universal HPV vaccination programme in England: protocol for a co‐design study. BMJ Open. 2022;12(4):e062050. 10.1136/bmjopen-2022-062050 PMC8987754 35387837

[55] Moran VH , Ceballos‐Rasgado M , Fatima S , et al. Participatory action research to co‐design a culturally appropriate COVID‐19 risk communication and community engagement strategy in rural Pakistan. Front Public Health. 2023;11:1160964. 37168074 10.3389/fpubh.2023.1160964 PMC10166109

[56] Jama A , Appelqvist E , Kulane A , et al. Design and implementation of tailored intervention to increase vaccine acceptance in a Somali community in Stockholm, Sweden—based on the Tailoring Immunization Programmes approach. Public Health Pract. 2022;4:100305. 10.1016/j.puhip.2022.100305 PMC9773050 36570400

[57] Verbiest MEA , Corrigan C , Dalhousie S , et al. Using codesign to develop a culturally tailored, behavior change mHealth intervention for indigenous and other priority communities: a case study in New Zealand. Transl Behav Med. 2018;9(4):720‐736. 10.1093/tbm/iby093 30388262

[58] Jackson C , Bedford H , Cheater FM , et al. Needles, Jabs and Jags: a qualitative exploration of barriers and facilitators to child and adult immunisation uptake among Gypsies, Travellers and Roma. BMC Public Health. 2017;17(1):254. 28288596 10.1186/s12889-017-4178-y PMC5348901

[59] Bell S , Saliba V , Ramsay M , Mounier‐Jack S . What have we learnt from measles outbreaks in 3 English cities? A qualitative exploration of factors influencing vaccination uptake in Romanian and Roma Romanian communities. BMC Public Health. 2020;20(1):381. 32293379 10.1186/s12889-020-8454-x PMC7092501

[60] Harmsen IA , Bos H , Ruiter RAC , et al. Vaccination decision‐making of immigrant parents in the Netherlands; a focus group study. BMC Public Health. 2015;15:1229. 26654538 10.1186/s12889-015-2572-x PMC4676170

[61] Salad J , Verdonk P , de Boer F , Abma TA . “A Somali girl is Muslim and does not have premarital sex. Is vaccination really necessary?” A qualitative study into the perceptions of Somali women in the Netherlands about the prevention of cervical cancer. Int J Equity Health. 2015;14:68. 26293806 10.1186/s12939-015-0198-3 PMC4546144

[62] Brockmann SO , Wjst S , Zelmer U , et al. ÖGD‐Initiative zur Verbesserung der Durchimpfung bei Asylsuchenden. Bundesgesundheitsblatt Gesundheitsforschung Gesundheitsschutz. 2016;59(5):592‐598. 27072499 10.1007/s00103-016-2335-6

[63] Mellou K , Silvestros C , Saranti‐Papasaranti E , et al. Increasing childhood vaccination coverage of the refugee and migrant population in Greece through the European programme PHILOS, April 2017 to April 2018. Euro Surveill. 2019;24(27):1800326. 31290391 10.2807/1560-7917.ES.2019.24.27.1800326 PMC6628755

[64] Taylor A , Radford G , Calia C . Review: cultural adaptations to psychosocial interventions for families with refugee/asylum‐seeker status in the United Kingdom—a systematic review. Child Adolescent Mental Health. 2023;28(2):241‐257. 35195944 10.1111/camh.12547

[65] Barrera, Jr. M , Castro FG , Strycker LA , Toobert DJ . Cultural adaptations of behavioral health interventions: a progress report. J Consult Clin Psychol. 2013;81(2):196‐205. 22289132 10.1037/a0027085 PMC3965302

[66] Lau LS , Rodgers G . Cultural competence in refugee service settings: a scoping review. Health Equity. 2021;5(1):124‐134. 33778315 10.1089/heq.2020.0094 PMC7990563

[67] Khatri RB , Assefa Y . Access to health services among culturally and linguistically diverse populations in the Australian universal health care system: issues and challenges. BMC Public Health. 2022;22(1):880. 35505307 10.1186/s12889-022-13256-z PMC9063872

[68] WHO Regional Office for Europe . *Migration and Health: Enhancing Intercultural Competence and Diversity Sensitivity*. WHO Regional Office for Europe; 2020.

[69] Cargo M , Mercer SL . The value and challenges of participatory research: strengthening its practice. Annu Rev Public Health. 2008;29(1):325‐350. 18173388 10.1146/annurev.publhealth.29.091307.083824

[70] Reason P , Torbert W . The action turn: toward a transformational social science. Concepts Transform. 2001;6(1):1‐37.

[71] Public Health England . *Community Champions: A Rapid Scoping Review of Community Champion Approaches for the Pandemic Response and Recovery*. Public Health England; 2021.

[72] NHS England . *Working in Partnership With People and Communities: Statutory Guidance*. NHS England; 2022.

[73] Voice4Change England . *Funding for Black, Asian and Other Minority Ethnic Communities: Bridging the Gap in Funding for the BAME Voluntary and Community Sector*. Voice4Change England; 2015.

[74] Charity So White . *Racial Injustice in the COVID‐19 Response: A Live Position Paper Online*; Charity So White; 2020.

[75] The Ubele Initiative . *Impact of COVID‐19 on BAME community and voluntary organisations: final report of the surveys conducted between 19 March and 4 April 2020*; 2020.

[76] Hargrave R . *Covid Fund for BAME Charities was Seven‐Times Oversubscribed*. Civil Society; 2022.

[77] Ten Years' Time . *Racial Justice & Social Transformation: How Funders can Act*. Ten Years' Time; 2022.

[78] Gray RE , Fitch M , Davis C , Phillips C . Challenges of participatory research: reflections on a study with breast cancer self‐help groups. Health Expect. 2000;3(4):243‐252. 11281935 10.1046/j.1369-6513.2000.00100.x PMC5060116

[79] Roura M , Dias S , LeMaster JW , MacFarlane A . Participatory health research with migrants: opportunities, challenges, and way forwards. Health Expect. 2021;24(2):188‐197. 33528082 10.1111/hex.13201 PMC8077110

[80] Bécares L , Shaw RJ , Katikireddi SV , et al. Racism as the fundamental cause of ethnic inequities in COVID‐19 vaccine hesitancy: a theoretical framework and empirical exploration using the UK Household Longitudinal Study. SSM Population Health. 2022;19:101150. 35765366 10.1016/j.ssmph.2022.101150 PMC9225926

[81] Streuli S , Lewis T . Shifting priorities and neoliberal ideologies in refugee health intervention design in the US. Med Anthropol. 2022;41(4):488‐502. 35394891 10.1080/01459740.2022.2053965

